# Palmitoylethanolamide Ameliorates Carbon Tetrachloride-Induced Liver Fibrosis in Rats

**DOI:** 10.3389/fphar.2018.00709

**Published:** 2018-07-13

**Authors:** Masatsugu Ohara, Shunsuke Ohnishi, Hidetaka Hosono, Koji Yamamoto, Qingjie Fu, Osamu Maehara, Goki Suda, Naoya Sakamoto

**Affiliations:** ^1^Department of Gastroenterology and Hepatology, Hokkaido University Graduate School of Medicine, Sapporo, Japan; ^2^Department of Pathophysiology and Therapeutics, Faculty of Pharmaceutical Sciences, Hokkaido University, Sapporo, Japan

**Keywords:** palmitoylethanolamide, carbon tetrachloride, liver fibrosis, hepatic stellate cells (HSCs), transforming growth factor-β, smad

## Abstract

**Background:** Liver fibrosis is a complex inflammatory and fibrogenic process, and the progression of fibrosis leads to cirrhosis. The only therapeutic approaches are the removal of injurious stimuli and liver transplantation. Therefore, the development of anti-fibrotic therapies is desired. Palmitoylethanolamide (PEA) is an endogenous fatty acid amide belonging to the *N*-acylethanolamines family and contained in foods such as egg yolks and peanuts. PEA has therapeutic anti-inflammatory, analgesic, and neuroprotective effects. However, the effects and roles of PEA in liver fibrosis remain unknown. Here we investigated the therapeutic effects of PEA in rats with liver fibrosis.

**Methods:** We conducted *in vitro* experiments to investigate the effects of PEA on the activation of hepatic stellate cells (HSCs, LX-2). Liver fibrosis was induced by an intraperitoneal injection of 1.5 mL/kg of 50% carbon tetrachloride twice a week for 4 weeks. Beginning at 3 weeks, PEA (20 mg/kg) was intraperitoneally injected thrice a week for 2 weeks. Then rats were sacrificed and we performed histological and quantitative reverse-transcription polymerase chain reaction analyses.

**Results:** The expression of α-smooth muscle actin (SMA) induced by transforming growth factor (TGF)-β1 in HSCs was significantly downregulated by PEA. PEA treatment inhibited the TGF-β1-induced phosphorylation of SMAD2 in a dose-dependent manner, and upregulated the expression of *SMAD7*. The reporter gene assay demonstrated that PEA downregulated the transcriptional activity of the SMAD complex upregulated by TGF-β1. Administration of PEA significantly reduced the fibrotic area, deposition of type I collagen, and activation of HSCs and Kupffer cells in rats with liver fibrosis.

**Conclusion:** Activation of HSCs was significantly decreased by PEA through suppression of the TGF-β1/SMAD signaling pathway. Administration of PEA produced significant improvement in a rat model of liver fibrosis, possibly by inhibiting the activation of HSCs and Kupffer cells. PEA may be a potential new treatment for liver fibrosis.

## Introduction

Liver fibrosis is a complex inflammatory and fibrogenic process that results from chronic liver injury (Zhang et al., 2016). It is characterized by the excessive deposition of extracellular matrix proteins including collagen, and by activation of HSCs and Kupffer cells (Friedman, 2008; Tacke and Zimmermann, 2014; van Dijk et al., 2015). HSCs, once activated, are responsible for liver fibrosis (Xu et al., 2005). A significant increase in TGF-β expression is observed in the activated HSCs (Yoshida et al., 2014). TGF-β family members (TGF-β1, -β2, and -β3) are induced and activated in a variety of fibrotic diseases (Govinden and Bhoola, 2003; Xu et al., 2014). The progression of liver fibrosis leads to cirrhosis, a condition characterized by distortion of the normal liver architecture, the formation of septae/nodules, portal hypertension, and eventually tumor formation (Han et al., 2004; van Dijk et al., 2015). The only therapeutic approaches are removal of the injurious stimuli and liver transplantation, which is limited by organ shortages, high associated expenses, and the need for lifelong immunosuppressive medications (Campana and Iredale, 2017; Wang et al., 2017). Therefore, development of anti-fibrotic therapies is a major area of unmet clinical need.

Palmitoylethanolamide is an endogenous fatty acid amide belonging to the *N*-acylethanolamines (NAEs) family, and contained in foods such as egg yolks, peanuts, and soy seeds (Kilaru et al., 2007; Esposito and Cuzzocrea, 2013; Impellizzeri et al., 2015). PEA is synthesized “on-demand” during a variety of inflammatory disease states and produces marked protective properties for inflammatory responses and pruritus (Re et al., 2007; Wang et al., 2014). Furthermore, PEA has therapeutic analgesic and neuroprotective effects, acting at different molecular targets such as tumor necrosis factor (TNF)-α, vascular endothelial growth factor (VEGF), and intercellular adhesion molecules (ICAM)-1 (Hoareau et al., 2006; Esposito and Cuzzocrea, 2013; Wang et al., 2014; Paterniti et al., 2015). PEA reduces lung inflammation in mice with pulmonary fibrosis (Di Paola et al., 2016), and is able to modulate activation of one or more members of the PPAR family of nuclear receptors and/or a cannabinoid CB2-like receptor (Di Paola et al., 2016). In addition, PPARα is required for the anti-inflammatory actions of PEA (Lo Verme et al., 2005), and presence of PPARα mRNA in HSCs (Miyahara et al., 2000) and suppressive effect of PPARα agonist Wy-14,643 on liver fibrosis (Ip et al., 2004) have been reported. However, the effects and roles of PEA in liver fibrosis remain unknown. The aim of this study was to examine the effects of PEA on liver fibrosis in rats and to investigate its underlying mechanisms.

## Materials and Methods

### Reagents

Palmitoylethanolamide, MK886, a PPARα antagonist, and GW6471, a PPARα antagonist, were purchased from Cayman Chemical (Ann Arbor, MI, United States). PEA was dissolved in dimethyl sulfoxide (Wako Pure Chemical Industries, Osaka, Japan) for the *in vitro* experiments, and dissolved in a vehicle composed of Tween 80 (Kanto Chemical, Tokyo, Japan), dimethyl sulfoxide, and phosphate-buffered saline (PBS, Life Technologies, Carlsbad, CA, United States) (1:0.5:18.5 by volume) for the *in vivo* experiments.

### Cell Culture

LX-2 cells, immortalized human HSCs, were purchased from Merck Millipore (Billerica, MA, United States). In all experiments, the cells were subjected to no more than 15 cell passages. The cells were cultured in Dulbecco’s modified Eagle’s medium (Thermo Fisher Scientific, Waltham, MA, United States) containing 2% fetal bovine serum (Moregate Biotech, Bulimba, QLD, Australia), 100 U/mL of penicillin, and 100 μg/mL of streptomycin (Wako Pure Chemical Industries), and maintained at 37°C in a humidified atmosphere of 5% CO_2_. The medium was changed every other day. Human embryonic kidney cells (HEK293, provided by the RIKEN BioResource Center, Tsukuba, Japan) or their derivatives, which were stably transfected with the human Toll-like receptor (TLR) 4a, MD2, and CD14 genes (293/hTLR4A-MD2-CD14; InvivoGen, San Diego, CA, United States), were cultured in Dulbecco’s modified Eagle’s medium containing 10% fetal bovine serum, 100 U/mL of penicillin, and 100 μg/mL of streptomycin. 293/hTLR4A-MD2-CD14 cells were activated by lipopolysaccharide (LPS; Sigma-Aldrich, St. Louis, MO, United States) treatment.

### Western Blot Analysis

To investigate the phosphorylation of SMAD2 and expression of α-SMA, LX-2 cells were plated into six-well plates (2 × 10^5^ cells/well, Corning, Corning, NY, United States) and cultured. The next day, the culture medium was changed to a medium containing PEA or dimethyl sulfoxide, and the cells were incubated for an additional 30 min or 1 h. Then cells were treated with 2.0 ng/mL of TGF-β1 (PeproTech, Rocky Hill, NJ, United States) for 30 min, 24 h, and 72 h, and washed with ice-cold PBS. Cell lysates were prepared using a radio-immunoprecipitation assay (RIPA) buffer containing 50 mM Tris-HCl (pH8.0), 150 mM NaCl, 0.5% (w/v) sodium deoxycholate, 0.1% (w/v) sodium dodecyl sulfate (SDS), 1.0% (w/v) NP-40 substitute, and Protease/Phosphatase Inhibitor Cocktail (Cell Signaling Technology, Beverly, MA, United States). Equal amounts of cellular protein extracts were diluted in a 4 × Laemmli sample buffer (Bio-Rad, Hercules, CA, United States). The samples were heated at 95°C for 5 min and then subjected to SDS-polyacrylamide gel electrophoresis (SDS-PAGE, Bio-Rad). The separate proteins were transferred to Immobilon-P polyvinylidene difluoride (PVDF) membranes (Merck Millipore), which were subsequently incubated in tris buffered saline with 0.05% Tween 20 (Wako Pure Chemical Industries) consisting of a 5% PhosphoBLOCKER blocking reagent (Cell Biolabs, San Diego, CA, United States) at room temperature for 60 min. The membranes were probed with primary antibodies for phospho-SMAD2 (1:2000; Cell Signaling Technology), SMAD2/3 (1:2000; Cell Signaling Technology), α-SMA (1:1000; Abcam, Cambridge, United Kingdom) and bound antibodies were detected with peroxidase AffiniPure Goat Anti-Mouse IgG (H + L) (1:10,000; Jackson ImmunoResearch, West Grove, PA, United States) or peroxidase AffiniPure Goat Anti-Rabbit IgG (H + L) (1:10,000; Jackson ImmunoResearch), and visualized and photographed using ECL Prime detection reagent (GE Healthcare, Chicago, IL, United States). The blots were analyzed using ImageQuant LAS-4000 (Fujifilm, Tokyo, Japan).

### Plasmids

cDNAs of TGF-β receptor 1 (TGFβR-1) and SMAD2 were obtained by RT-PCR and the resulting fragments were cloned into pCI-neo-HA(c) and pCMV-3 × FLAG. Active mutants of TGFβR-1 (T204D) (Wieser et al., 1995) and SMAD2-2D (S465D/S467D) (Souchelnytskyi et al., 1997) were consecutively generated using a PCR-based method.

### Transient Transfection and Reporter Gene Assay

LX-2 cells and HEK293 cells were plated into 24-well plates (Corning) containing 500 μL of culture medium (4.0 × 10^4^ cells/well and 1.25 × 10^5^ cells/well, respectively). After incubation for 24 h at 37°C, cells were transfected with 25 ng of luciferase plasmid DNA with 25 ng of Renilla pGL4.74(hRluc/TK) vector (Promega, Madison, WI, United States) as an internal control, and 500 ng of plasmid DNA containing three copies of a SMAD binding element that drove the transcription of the luciferase reporter gene [pGL4.48(luc2P/SBE/Hygro), Promega], using Lipofectamine^®^ LTX (Life Technologies). After 24 h of incubation at 37°C, the cells were treated with 1.0 ng/mL of TGF-β1 for 3 h; a reporter gene assay was performed using the Dual Luciferase Reporter Assay System (Promega). Luminescence intensity was measured using a GloMax^®^-Multi Detection System (Promega) according to manufacturer’s instructions. Transcription activity was normalized according to the Renilla luciferase activity. These experiments were performed in triplicate.

### Immunofluorescent Staining

LX-2 cells were plated into a eight-well coverglass chamber (Asahi Glass, Tokyo, Japan), and pretreated with PEA for 1 h. Then, the cells were incubated with 2.0 ng/mL of TGF-β1 for 24 h. After incubation, the cells were washed with PBS, and then fixed in 100% methanol (Wako Pure Chemical Industries) for 15 min at 4°C. The cells were blocked with 2% human serum albumin (The Chemo-Sero-Therapeutic Research Institute, Kumamoto, Japan) in PBS for 1 h at 4°C. A primary antibody against α-SMA (1:250; Abcam) was incubated for 2 h at 4°C, and then the cells were incubated with a fluorescein-labeled secondary antibody (1:2000; Cell Signaling Technology) for 1 h. Samples were imaged using a fluorescence microscopy (Olympus, Tokyo, Japan). Fluorescent intensities were analyzed using ImageJ (NIH, Bethesda, MD, United States). At least 10 cells were included in the analysis for each condition.

### Animals

This study was carried out in accordance with the recommendations of the Animal Care Unit and Use Committees of Hokkaido University. The protocol was approved by the Animal Care Unit and Use Committees of Hokkaido University. Six-week-old male Sprague-Dawley rats were procured from Japan SLC (Hamamatsu, Japan), and three rats were housed per cage in a temperature-controlled room (24°C) on a 12-h/12-h light/dark cycle. All rats had *ad libitum* access to standard chow and water.

### Induction of Liver Fibrosis and PEA Treatment

**FIGURE 1:**
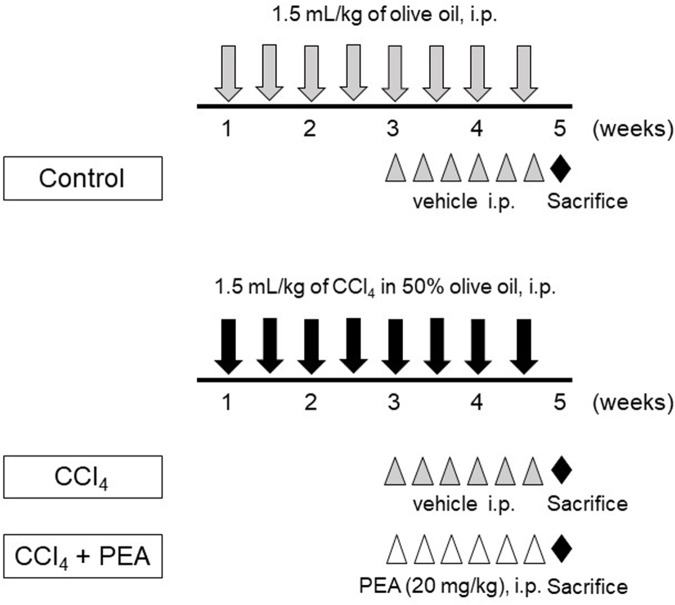
Experimental protocol for carbon tetrachlroide (CCl_4_)-induced liver fibrosis. Rats received intraperitoneal (i.p.) injection of 1.5 mL/kg of CCl_4_ in 50% olive oil twice a week for 4 weeks. Palmitoylethanolamide (PEA, 20 mg/kg) was i.p. injected thrice a week beginning at 3 weeks. All rats were sacrificed at 5 weeks.

Liver fibrosis was induced by an intraperitoneal injection of 1.5 mL/kg of 50% carbon tetrachloride (CCl_4_, Wako Pure Chemical Industries) in olive oil twice a week for 4 weeks (**Figure 1**). The control group rats were injected with olive oil alone. PEA (20 mg/kg) was injected intraperitoneally thrice a week after 2 weeks of CCl_4_ treatment. The dose of PEA was based on a previously reported study (Richter et al., 2016).

### Histological Examination

The rats were sacrificed after 4 weeks of CCl_4_ treatment. The left lobe of the liver was removed, fixed in 40 g/L of formaldehyde saline, embedded in paraffin, and cut into 5-μm sections. Tissue sections were stained with Hematoxylin and Eosin (H&E) and Masson’s trichrome. We photographed 10 random fields on a section from each rat, and calculated the blue-stained area from the entire liver cross-sectional area (%, ×100) with a digital image analyzer (WinROOF; Mitani, Co., Fukui, Japan).

### Immunohistochemical Examination

The tissue sections were stained with anti-rat type I collagen antibody (1:100,000; LSL, Tokyo, Japan) for 60 min at room temperature. To assess HSC activation, the tissue sections were stained with anti-rat α-SMA antibody (1:800, Thermo Fisher Scientific) for 30 min at room temperature. To assess the infiltration of Kupffer cells, the tissue sections were stained with anti-rat CD68 monoclonal antibody (1:50; AbD Serotec, Kidlington, United Kingdom) for 40 min at room temperature. We photographed 10 random fields on a section from each rat, and measured the stained areas from the entire liver cross-sectional area using a digital image analyzer (WinROOF).

### RNA Isolation and Quantitative RT-PCR

Total RNA of cultured cells or the rat liver was extracted using the RNeasy Mini Kit (Qiagen, Hilden, Germany), and 1 μg of the total RNA was reverse-transcribed into cDNA using the PrimeScript RT reagent Kit (Takara Bio, Kusatsu, Japan). PCR was performed using a 25-μL reaction mixture that contained 1 μL of cDNA and 12.5 μL Platinum SYBR Green PCR Mix (Life Technologies). β-actin messenger RNA that was amplified from the same samples served as an internal control. After initial denaturation at 95°C for 2 min, we used a two-step cycle procedure (denaturation at 95°C for 15 s, annealing and extension at 60°C for 1 min) for 40 cycles in a 7700 Sequence Detector (Applied Biosystems, Foster City, CA, United States). Gene expression levels were determined using the comparative threshold cycle (ΔΔCt) method with β-actin used as an endogenous control. Data were analyzed with Sequences Detection Systems software (Applied Biosystems). Primer sequences are shown in **Table 1**.

**Table 1:** Primer sequences.

human TNF-α F	CAGCCTCTTCTCCTTCCTGA
human TNF-α R	GCCAGAGGGCTGATTAGAGA
human α-SMA F	CCGACCGAATGCAGAAGGA
human α-SMA R	ACAGAGTATTTGCGCTCCGAA
human collagen1a1 F	GCTCCTCTTAGGGGCCACT
human collagen1a1 R	CCACGTCTCACCATTGGGG
human SMAD7 F	TCCTGCTGTGCAAAGTGTTC
human SMAD7 R	TTGTTGTCCGAATTGAGCTG
human β-actin F	CCAACCGCGAGAAGATGA
human β-actin R	CCAGAGGCGTACAGGGATAG
rat α-SMA F	GACACCAGGGAGTGATGGTT
rat α-SMA R	GTTAGCAAGGTCGGATGCTC
rat collagen1a1 F	GATGGCTGCACGAGTCACAC
rat collagen1a1 R	ATTGGGATGGAGGGAGTTTA
rat TGF-β F	CTGCTGACCCCCACTGATAC
rat TGF-β R	AGCCCTGTATTCCGTCTCCT
rat TNF-α F	GGCTCCCTCTCATCAGTTCCA
rat TNF-α R	CGCTTGGTGGTTTGCTACGA
rat TIMP1 F	GACCACCTTATACCAGCGTT
rat TIMP1 R	GTCACTCTCCAGTTTGCAAG
rat MMP9 F	TTATTGTGAGCATCCCTAGGG
rat MMP9 R	AGTGTCCGAGGAAGATACTTG
rat PPARα F	AATCCACGAAGCCTACCTGA
rat PPARα R	GTCTTCTCAGCCATGCACAA
rat CD68 F	TCACAAAAAGGCTGCCACTCTT
rat CD68 R	TCGTAGGGCTTGCTGTGCTT
rat MCP-1 F	CTGTCTCAGCCAGATGCAGTTAA
rat MCP-1 R	AGCCGACTCATTGGGATCAT
rat LBP F	AACATCCGGCTGAACACCAAG
rat LBP R	CAAGGACAGATTCCCAGGACTGA
rat β-actin F	AAGATGACCCAGATCATGTT
rat β-actin R	TTAATGTCACGCACGATTTC


*F, forward; R, reverse.*

### Statistical Analysis

Data are expressed as mean ± standard deviation (*SD*). Parameters among the groups were compared by one-way ANOVA, followed by Tukey’s test. The difference was considered significant at *p* < 0.05. All analyses were performed using the GraphPad Prism, version 7 (GraphPad software, San Diego, CA, United States).

## Results

### PEA Suppresses LPS-Induced Inflammatory Reaction and TGF-β1-Induced Activation of HSCs *in Vitro*

**FIGURE 2:**
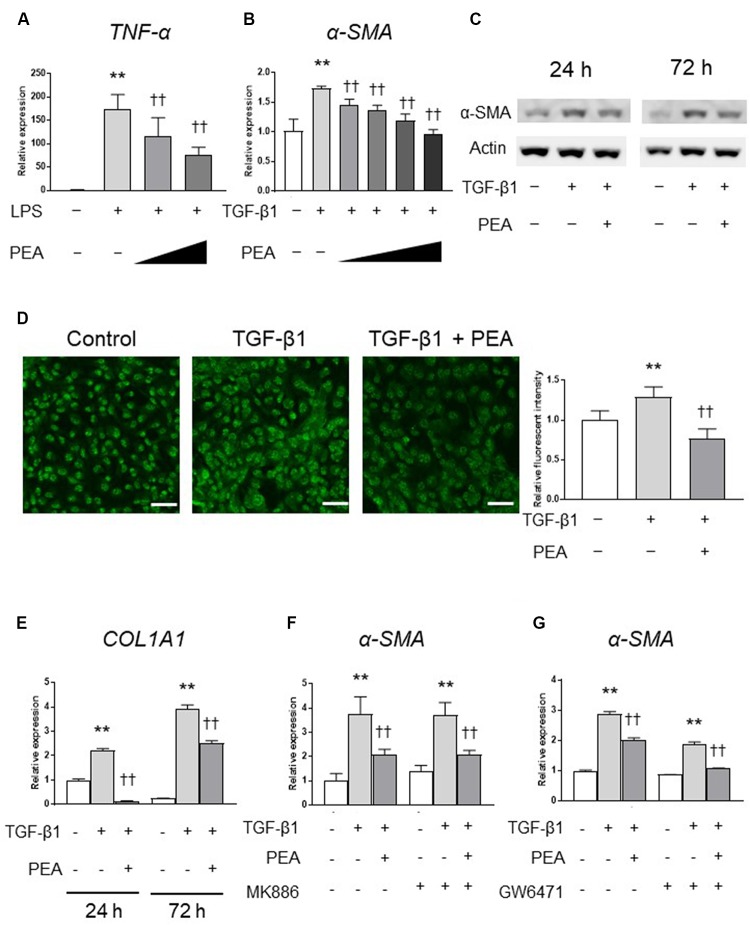
Effect of palmitoylethanolamide (PEA) on inflammatory reactions and fibrogenic responses *in vitro*. **(A)** PEA (0.1, 1.0 μM) was added to cultured 293/hTLR4A-MD2-CD14 cells with 2.5 ng/mL lipopolysaccharide (LPS). Total RNA was isolated 3 h after LPS administration, and the expression of tumor necrosis factor (*TNF*)-α was investigated by quantitative reverse-transcription polymerase chain reaction (qRT-PCR). **(B)** PEA (1.0, 5.0, 10.0, and 20.0 μM) was added to LX-2 cells with 2.0 ng/mL transforming growth factor (TGF)-β1. Total RNA was isolated 24 h after TGF-β1 administration, and the expression of α-smooth muscle actin (*SMA*) was investigated by qRT-PCR. **(C)** PEA (10 μM) was added to LX-2 cells with 2.0 ng/mL TGF-β1. Total protein was isolated 24 or 72 h after TGF-β1 administration and the expression of α-SMA was investigated by Western blotting. **(D)** Immunofluorescent staining with α-SMA (green) in LX-2 cells 24 h after TGF-β1 administration. Scale bars: 20 μm. **(E)** PEA (10 μM) was added to LX-2 cells with 2.0 ng/mL TGF-β1. Total RNA was isolated 24 and 72 h after TGF-β1 administration and the expression of collagen1a1 (*COL1A1*) was investigated by qRT-PCR. **(F)** LX-2 cells were treated with PEA (10 μM) followed by TGF-β1 for 24 h with or without MK886 (10 μM) treatment. **(G)** LX-2 cells were treated with PEA (10 μM) followed by TGF-β1 for 24 h with or without GW6471 (2 μM) treatment. The values are the mean ± standard deviation (*n* = 3). ^∗∗^*p* < 0.01 versus the Control. ^††^*p* < 0.01 versus TGF-β1.

We first investigated the anti-inflammatory effect of PEA in 293/hTLR4A-MD2-CD14 cells. Treatment with LPS significantly upregulated the expression of *TNF-α*, and PEA significantly and dose-dependently decreased the expression of *TNF-α* (**Figure 2A**). We next examined whether PEA suppresses TGF-β1-induced activation of LX-2 cells. Treatment with TGF-β1 significantly upregulated the expression of *α-SMA*, and PEA significantly downregulated the expression of *α-SMA* in a dose-dependent manner (**Figure 2B**). Similarly, Western blotting and immunofluorescent staining demonstrated that PEA decreased the protein levels of α-SMA that were induced by TGF-β1 (**Figures 2C,D**, respectively). Furthermore, treatment with TGF-β1 significantly increased the expression of collagen1a1 (*COL1A1*) in LX-2 cells, and PEA significantly decreased the expression of *COL1A1* (**Figure 2E**). However, the inhibitory effect of PEA on activation of LX-2 cells were not canceled by PPARα antagonist MK886 and GW6471 (**Figures 2F,G**, respectively). These results suggest that PEA suppresses LPS-induced inflammatory reaction and TGF-β1-induced fibrogenic reaction. In addition, the mechanisms of the inhibitory effect on HSC activation are independent of PPARα.

### PEA Suppresses Phosphorylation of SMAD2 and Upregulates *SMAD7*

**FIGURE 3:**
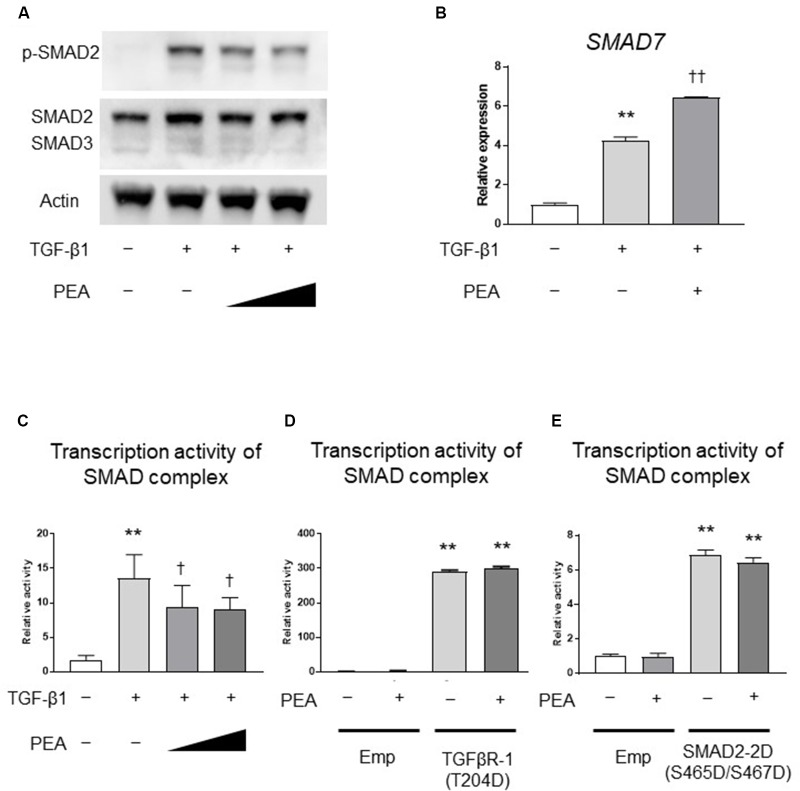
Effect of PEA on TGF-β/SMAD signaling pathways. **(A)** LX-2 cells were treated with TGF-β1 (2.0 ng/mL) for 30 min after pretreatment with PEA (10 and 20 μM) for 30 min, and the expression of phosphorylated SMAD2 (p-SMAD2) was investigated by Western blotting. **(B)** Effect of PEA (10 μM) on mRNA expression of *SMAD7* in LX-2 cells treated with TGF-β1 (2.0 ng/mL) for 30 min. **(C)** Effect of PEA (10 and 20 μM) on transcription activity of SMAD complex in LX-2 cells treated with TGF-β1 (1.0 ng/mL) for 3 h. **(D)** Effect of PEA (20 μM) on transcription activity of SMAD complex in LX-2 cells with overexpression of TGFβR-1 (T204D). **(E)** Effect of PEA (20 μM) on transcription activity of SMAD complex in LX-2 cells with overexpression of SMAD2-2D (S465D/S467D). The values are the mean ± standard deviation (*n* = 3). ^∗∗^*p* < 0.01 versus the Control. ^†^*p* < 0.05, ^††^*p* < 0.01 versus TGF-β1.

We next investigated whether PEA affects the TGF-β/SMAD pathway. Western blotting demonstrated that TGF-β1 treatment induced the phosphorylation of SMAD2, and PEA treatment inhibited the TGF-β1-induced phosphorylation of SMAD2 in a dose-dependent manner (**Figure 3A**). In addition, treatment with TGF-β1 significantly increased the expression of *SMAD7*, an inhibitor of the SMAD pathway, and PEA treatment further increased the expression of *SMAD7* (**Figure 3B**). The reporter gene assay demonstrated that TGF-β1 markedly upregulated the transcription activity of the SMAD complex, and it was significantly downregulated by PEA (**Figure 3C**). Overexpression of TGFβR-1 (T204D), a constitutively active form of TGFβR-1, significantly upregulated the transcription activity of the SMAD complex, but PEA did not suppress luciferase activity (**Figure 3D**). Furthermore, overexpression of SMAD2-2D (S465D/S467D), a constitutively active form of SMAD2, also significantly elevated the transcription activity of the SMAD complex, but PEA did not suppress the luciferase activity (**Figure 3E**). These results suggest that PEA suppresses an early step of TGF-β/SMAD pathway in LX-2 cells.

### PEA Suppresses Liver Fibrosis in Rats

**FIGURE 4:**
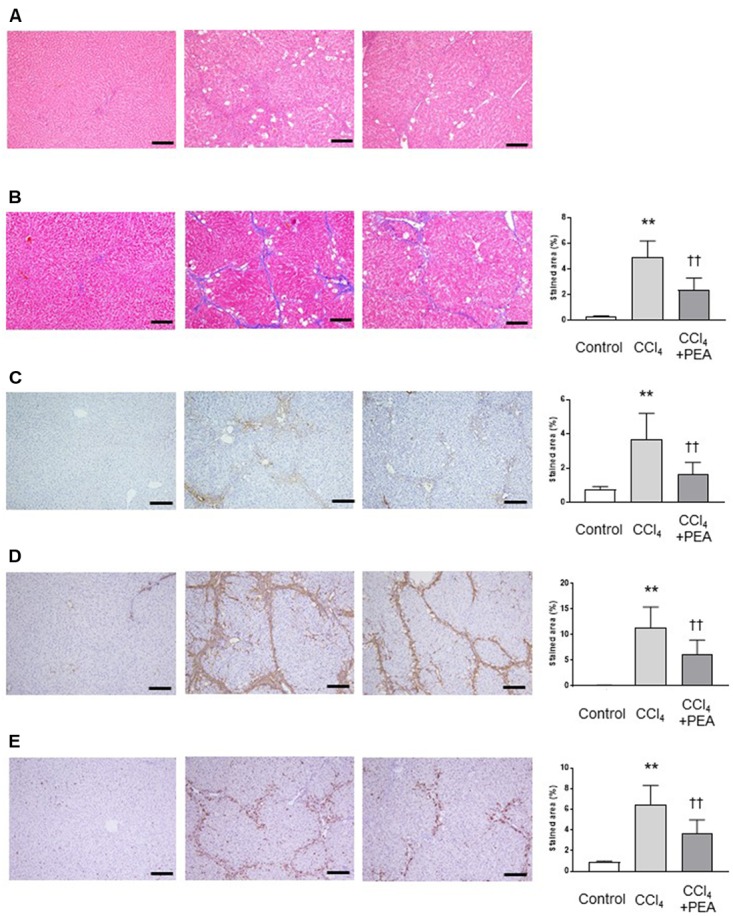
Effect of PEA on liver fibrosis in rats. **(A)** Hematoxylin and eosin staining. **(B)** Masson’s trichrome staining. Fibrotic area was stained blue and measured from the entire liver cross-sectional area. **(C)** Expression of type I collagen. **(D)** α-SMA expression **(E)** CD68 expression. The stained areas were measured from the entire liver cross-sectional area. Scale bars: 200 μm. The values are the mean ± standard deviation of (*n* = 6 in Control group, *n* = 14 in CCl_4_ group, and *n* = 14 in CCl_4_ + PEA group). ^∗∗^*p* < 0.01 versus Control group. ^††^*p* < 0.01 versus CCl_4_ group.

We investigated the effects of PEA on liver fibrosis in rats. In H&E staining, the thick fibrotic septa and pseudolobular formation were more extensive in the CCl_4_ group compared to the PEA group (**Figure 4A**). Masson’s trichrome staining demonstrated that fiber accumulation induced by CCl_4_ was significantly attenuated by administration of PEA for 2 weeks (**Figure 4B**). Consistent with this finding, the upregulated expression of type I collagen by CCl_4_ treatment was also attenuated by PEA administration (**Figure 4C**). The expression of α-SMA, a marker for HSC activation, was significantly increased in the CCl_4_ group; however, PEA administration significantly suppressed this increase (**Figure 4D**). The expression of CD68, a marker for Kupffer cells, was significantly increased in the CCl_4_ group; however, PEA administration decreased the infiltration of CD68-positive Kupffer cells (**Figure 4E**).

### Effects of PEA Administration on Gene Expressions in the Liver

**FIGURE 5:**
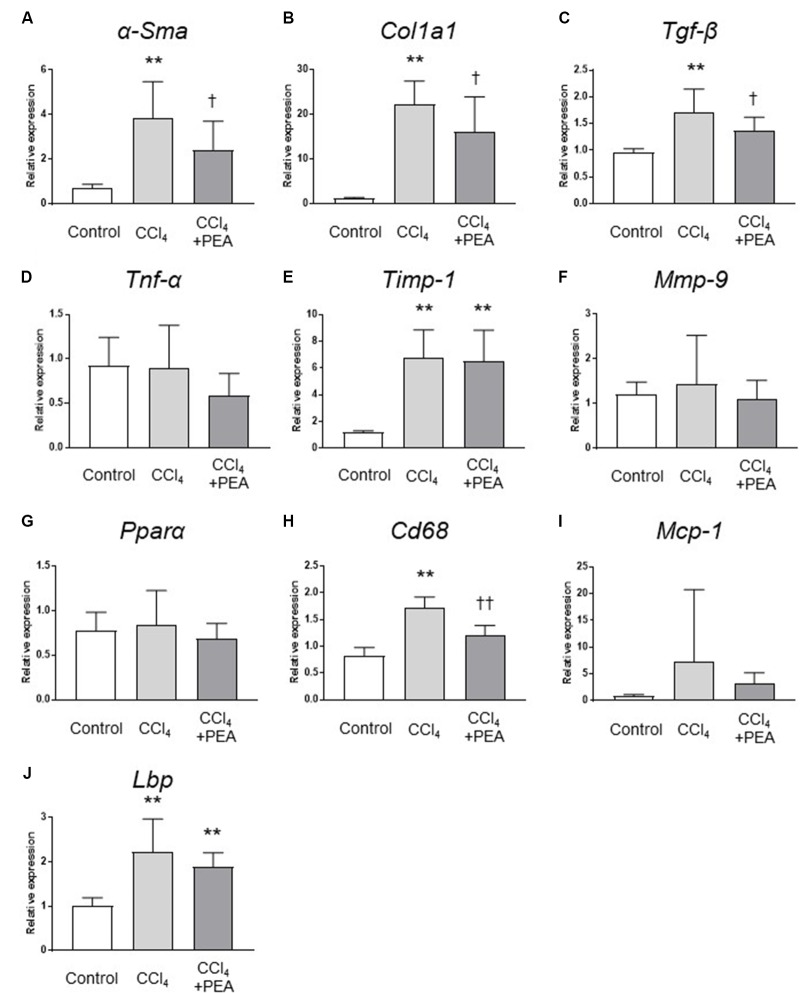
Gene expression analyses of the liver. qRT-PCR analyses of **(A)** α-smooth muscle actin (*α-Sma*), **(B)** collagen1a1 (*Col1a1*), **(C)** *Tgf-β*, **(D)** *Tnf-α*, **(E)** tissue inhibitor of metalloproteinases (*Timp*)*-1*, **(F)** matrix metalloproteinase (*Mmp*)*-9*, **(G)** peroxisome proliferator-activated receptor (*Ppar*)*α*, **(H)** *Cd68*, **(I)** monocyte chemoattractant protein (*Mcp*)*-1*, **(J)** lipopolysaccharide-binding protein (*Lbp*). The values are the mean ± standard deviation (*n* = 6 in Control group, *n* = 14 in CCl_4_ group, and *n* = 14 in CCl_4_ + PEA group). ^∗∗^*p* < 0.01 versus Control group. ^†^*p* < 0.05 versus CCl_4_ group. ^††^*p* < 0.01 versus CCl_4_ group.

We next examined the expression of fibrosis-related genes in the liver. CCl_4_ treatment significantly increased the expression of *α-Sma*, collagen1a1 (*Col1a1*), *Tgf-β*, and tissue inhibitor of metalloproteinases (*Timp*)*-1* (**Figures 5A–C,E**, respectively), and the expression of *α-Sma*, *Col1a1* and *Tgf-β* were significantly decreased by administration of PEA (**Figures 5A–C**). The expression of *Cd68* was significantly increased by CCl_4_; however, PEA significantly decreased the expression of *Cd68* (**Figure 5H**). The expressions of monocyte chemoattractant protein (*Mcp*)*-1* and *Lbp* tended to decrease following administration of PEA (**Figures 5I,J**, respectively). The expressions of matrix metalloproteinase (*Mmp*)*-9*, *Tnf-α* and *Pparα* were not affected by CCl_4_ treatment and PEA administration (**Figures 5D,F,G**, respectively).

## Discussion

In this study, we investigated the anti-fibrotic effects of PEA *in vitro* and *in vivo*. We found that (i) PEA suppressed LPS-induced inflammatory reaction *in vitro*, (ii) PEA suppressed TGF-β1-induced activation of cultured HSCs, and (iii) PEA administration ameliorated liver fibrosis in rats.

The therapeutic effects of PEA were previously reported in small animals with intestinal radiation injuries (Wang et al., 2014), uveitis (Impellizzeri et al., 2015), and cortical spreading depression (Richter et al., 2016). Proposed targets for PEA actions included a number of receptors, namely, cannabinoid receptors (Farquhar-Smith et al., 2002), transient receptor potential vanilloid type-1 (TRPV1) ion channels (Ambrosino et al., 2013), the orphan G protein-coupled receptor 55 (GPR55) (Pertwee, 2007), and PPARα (Lo Verme et al., 2005). PPARα is one of the main pharmacological targets of PEA action (LoVerme et al., 2005). On the other hand, PPARα is prominently expressed in hepatocytes (Ying-Ying and Lin, 2010), and PPARα ligands exert antifibrotic effects in rats with thioacetamide-induced liver cirrhosis (Toyama et al., 2004). Furthermore, PPARα agonists contrast inflammation and fibrosis in experimental models of steatohepatitis (Ip et al., 2004). However, in the present study, PEA-induced inactivation of LX-2 cells was not canceled by PPARα antagonists, suggesting that PEA attenuates activation of HSCs, independent of PPARα. Furthermore, Paterniti et al. (2015) demonstrated that PEA attenuates the degree of inflammation while preserving the blood–retinal barrier in rats with experimental diabetic retinopathy. This study revealed that PEA treatment reduces VEGF levels of the retina tissues in diabetic rats, and suggested that PEA may directly inhibit VEGF or may affect VEGF expression through TNF-α via its effects on VEGFR-2.

The molecular mechanisms of HSC activation were recently investigated (Huang et al., 2017; Senoo et al., 2017). TGF-β1 is known to be the most potent pro-fibrogenic cytokine in HSCs’ activation (Liu et al., 2006). We demonstrated that PEA inhibited the TGFβ-1-induced expression of fibrogenic genes, such as *α-SMA* and *COL1A1* in LX-2 cells. TGF-β regulates numerous cell signaling pathways (Doerks et al., 2002), and the TGF-β/SMAD signaling pathway is one of the key fibrogenic and inflammatory pathways in the liver (Gressner and Weiskirchen, 2006). It is well-known that TGF-β activates downstream mediators SMAD2 and SMAD3, and the TGF-β/SMAD pathway is negatively regulated by SMAD7 (Xu et al., 2016). Our *in vitro* study demonstrated that PEA inhibited phosphorylation of SMAD2 and upregulated *SMAD7* in LX-2 cells. In addition, PEA suppressed transcription activity of the SMAD complex, and expression of their downstream target genes and proteins. TGF-β1 binds TGFβR-2 with high affinity, which then recruits the lower-affinity TGFβR-1 (Hata and Chen, 2016). This induces the assembly of a complex that includes these receptors (Macias et al., 2015). Within the complex, TGFβR-2 subunits phosphorylate TGFβR-1 (Weiss and Attisano, 2013). Activated TGFβR-1 kinases recruit and phosphorylate SMAD proteins for signal transduction. Our *in vitro* study demonstrated that PEA did not suppress the transcription activity of the SMAD complex in HEK293 cells with overexpression of TGFβR-1 (T204D). Therefore, PEA may have suppressed the early steps of the TGF-β1/SMAD signaling pathway by interacting with TGFβR, or at the cell membrane.

In the present study, CCl_4_ was used to induce liver fibrosis. Liver fibrosis can be induced by one of the following approaches: (1) Chemical compounds and toxins. These agents cause direct injury to hepatocytes and trigger secondary inflammatory reaction in the liver, which in turn activate HSCs and result in fibrosis. Commonly used chemical agents include CCl_4_, thioacetamide, dimethyl nitrosamine, dioxin, sodium arsenate, and ethanol; (2) Special diet, such as choline-deficient, L-amino acid-defined, methionine-deficient diet, and high-fat diet; (3) Physical methods. Bile duct ligation creates obstruction of the extrahepatic bile duct; (4) Fibrosis induced by immune reaction; (5) Genetic modification (Zhou et al., 2014). Among above methods, bile duct ligation and CCl_4_ are well-validated models for fibrosis progression and resolution (Hernandez-Gea and Friedman, 2011). Therefore, we chose CCl_4_ to induce liver fibrosis in rats. This chemical changes the membrane permeability in liver mitochondria and plasm in various animals, and forms highly toxic free radicals, probably mediated by cytochrome p450 2E1 (Khan et al., 2015). Its repeated and prolonged treatment (≥4 weeks) causes severe liver disease, such as fibrosis and cirrhosis (Campana and Iredale, 2017). In liver fibrosis, the accumulation of extracellular matrix is a hallmark feature, and the activation of HSCs is a precursor of this phenomenon. In the normal liver, HSCs are quiescent and not fibrogenic (Senoo et al., 2017); however, the cells are activated by inflammatory processes of liver injury, such as the production of toxic cytokines and the recruitment of inflammatory cells (Bataller and Brenner, 2005). In the present study, we measured liver weight/body weight, and it was significantly increased by CCl_4_, and PEA treatment tended decrease the liver weight/body weight. The reason for this may be that it was the early stage of fibrosis with administration of CCl_4_ for 4 weeks (data are shown in Supplementary Materials). However, PEA administration suppressed CCl_4_-induced collagen deposition and activation of HSCs. In addition, PEA administration inhibited the expression of TGF-β in the liver. During liver injury, activated Kupffer cells secrete a large number of proinflammatory and fibrogenic mediators, which can drive HSC activation (Li et al., 2017) and are main targets of LPS, through TLR4 (Hernandez-Gea and Friedman, 2011). In the present study, PEA suppressed activation of 293/hTLR4A-MD2-CD14 cells, and inhibited the infiltration of Kupffer cells in rats with CCl_4_-induced liver injury. It has been reported that PEA has anti-inflammatory effect (Re et al., 2007), and PEA can attenuate LPS-induced inflammatory responses in the murine macrophage cell line RAW264.7 (Ross et al., 2000). Our *in vitro* experiment demonstrated that PEA has the anti-inflammatory effect in 293/hTLR4A-MD2-CD14 through LPS/TLR4 signal. Furthermore, macrophages are key-immune cells in the inflammatory process of liver fibrosis (Li et al., 2017), and responsible for liver cirrhosis induced by CCl_4_ (Muriel and Escobar, 2003). For other representative immune cells, it has been reported that neutrophils did not infiltrate in CCl_4_-induced liver fibrosis model (Campana and Iredale, 2017). Therefore, we evaluated the expression of CD68 in our *in vivo* experiment, and PEA would attenuate liver fibrosis by inhibiting activation of HSCs as well as Kupffer cells.

## Conclusion

PEA administration ameliorated fibrogenesis in a rat model of CCl_4_-induced liver fibrosis, possibly by attenuating the activation of HSCs and Kupffer cells. PEA administration may be a new therapeutic strategy for treating liver fibrosis, and should be investigated in other liver fibrosis models as well as human pathogenesis.

## Data Availability

The raw data supporting the conclusion of this manuscript will be made available by the authors, without undue reservation.

## Author Contributions

MO contributed to all experiments, data analysis, and manuscript writing. SO and NS contributed to conception and design, and final approval of the manuscript. HH, KY, QF, OM, and GS contributed to assembly of data and data analysis. All authors have read and approved the manuscript.

## Conflict of Interest Statement

GS has received honoraria from Bristol-Myers Squibb, MSD KK, and AbbVie. Naoya Sakamoto has received honoraria and research funding from Gilead Sciences, Bristol-Myers Squibb, MSD KK, Otsuka Pharm, Abbvie, Shionogi, and Takeda. The remaining authors declare that the research was conducted in the absence of any commercial or financial relationships that could be construed as a potential conflict of interest.

## Abbreviations

α-SMA: α-smooth muscle actin
CCl_4_: carbon tetrachloride; Col1a1, collagen1a1
HSC: hepatic stellate cell
LBP: lipopolysaccharide-binding protein
MMP: matrix metalloproteinase
PBS: phosphate-buffered saline
PEA: palmitoylethanolamide
PPAR: peroxisome proliferator-activated receptor
RT-PCR: reverse transcription-polymerase chain reaction
TGF-β: transforming growth factor-β
TGFβR: transforming growth factor-β receptor
TIMPs: tissue inhibitor of metalloproteinases
TNF-α: tumor necrosis factor-α
