# Supplementary material for: Palmitoylethanolamide Ameliorates Carbon Tetrachloride-Induced Liver Fibrosis in Rats
Source: Front Pharmacol. 2018 Jul 13;9:709. doi: 10.3389/fphar.2018.00709 (PMC6053486; doi:10.3389/fphar.2018.00709)

*Supplementary Material*

# Palmitoylethanolamide ameliorates carbon tetrachloride-induced liver fibrosis in rats


Masatsugu Ohara, Shunsuke Ohnishi*****, Hidetaka Hosono, Koji Yamamoto, Qingjie Fu,

Osamu Maehara, Goki Suda, and Naoya Sakamoto

*** Correspondence:** Dr. Shunsuke Ohnishi sonishi@pop.med.hokudai.ac.jp


## 1 Supplementary Data

Figure and Figure legend

Supplementary Figure 1.

Liver weight/body weight was measured. The values are the mean ± standard deviation of (n = 6 in Control group, n = 14 in $CCl_4$ group, and n =14 in $CCl_4$ + PEA group). ***p* < 0.01 versus Control group.

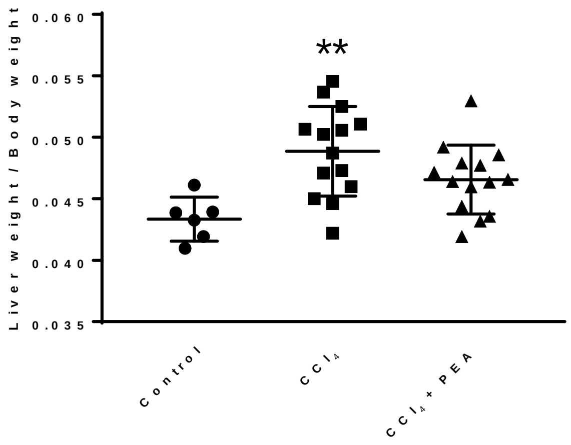

Supplement: Supplementary file 1 [file Image_1.PDF]
